# Short Communication: Evaluation of Intestinal Release of Butyric Acid from Sodium Butyrate Protected by Salts of Medium-Chain Fatty Acids in Broiler Chickens

**DOI:** 10.3390/ani12243525

**Published:** 2022-12-13

**Authors:** Meritxell Sadurní, Ana Cristina Barroeta, Cinta Sol, Mónica Puyalto, Lorena Castillejos

**Affiliations:** 1 Animal Nutrition and Welfare Service (SNiBA), Animal and Food Science Department, Faculty of Veterinary, Universitat Autònoma de Barcelona, 08193 Bellaterra, Spain; 2 Norel S.A., 28007 Madrid, Spain

**Keywords:** butyric acid, protected butyrate, medium-chain fatty acid, feed additive, gut health, intestinal release, broiler

## Abstract

**Simple Summary:**

Butyric acid is under investigation as a dietary supplement to improve gut health of broiler chickens. In fact, many studies have focused on the protection of butyric acid and its release throughout the intestine, promoting its beneficial effects in the entire gastrointestinal tract. Therefore, this study evaluated the intestinal release of butyric acid in broiler chickens by performing an in vivo approach. Broilers fed a diet supplemented with sodium butyrate protected by salts of medium-chain fatty acid containing a dye, brilliant blue. The blue color was then observed from jejunum and backward. Indeed, the released brilliant blue, and consequently, butyric acid, was quantified by intestinal sections observing that the major amount of butyric acid was delivered in the distal ileum. These results suggest that this in vivo approach enables the evaluation of the intestinal release of butyric acid from fat-protected sodium butyrate, showing a slow intestinal release of butyric acid in broiler chickens.

**Abstract:**

Butyric acid has received great attention as a feed additive to maintain or increase the gut integrity and health of broiler chickens. Particularly, the protection of butyrate is under research to allow slow intestinal release of butyric acid and to promote its beneficial effects throughout the intestine. This study evaluated in vivo the intestinal release of butyric acid from sodium butyrate protected by salts of medium-chain fatty acid in broilers. Brilliant blue was used as an inert marker, so it was included in the feed additive that broilers ingested for two days. The gastrointestinal tract was then colored in blue from jejunum and backward. Considering the digesta color of the broilers non-supplemented as blank, it allowed quantification of the amount of brilliant blue, and consequently, butyric acid delivered in the intestine from the protected feed additive. Few traces of butyric acid were released in the duodenum and proximal jejunum, whereas the major amount (45.9%) was delivered in the distal ileum (*p* < 0.001). These results suggest that this in vivo approach allows for evaluation of the intestinal delivery of butyric acid supplemented as protected sodium butyrate by medium-chain fatty acids, showing a gradual intestinal release of butyric acid in broiler chickens.

## 1. Introduction

The reduction of antibiotics use in poultry production has prompted increased investigation of feed additives with efficient properties to promote the gut health of broiler chickens, thereby supporting animal performance [1,2]. Butyric acid, a source of energy for enterocytes and with antibacterial effects, has received great attention as a dietary supplement for maintaining or enhancing gut health in broilers [3]. However, free butyric acid, as a short-chain fatty acid (SCFA), is naturally volatile and characterized by an unpleasant odor, and is also rapidly absorbed in the gastrointestinal tract (GIT) [4,5]. In this context, sodium butyrate is commonly utilized as a chemical salt derivative of free butyric acid to improve handling and bioavailability in the intestine [6,7]. Furthermore, protection of butyrate is under research to allow for the slow and gradual release of butyric acid throughout the GIT, increasing its availability [8]. Most common protective materials used are based on lipids [9,10]. Among them, medium-chain fatty acids (MCFA), even in salt form, may be an interesting strategy due to their possible synergistic effect on the intestinal barrier [11,12]. In fact, previous authors described improvements in gut morphological structure and antimicrobial effects by MCFA [13]. However, there is a lack of information on the intestinal release of butyric acid from protected forms. Indeed, to the best of the authors’ knowledge, few studies have evaluated in vivo release kinetics of protected feed additives, leaving gaps in their quantification [14,15]. Therefore, the aim of the present study was to evaluate the intestinal release of butyric acid from sodium butyrate protected by salts of MCFA in broiler chickens using a novel in vivo approach.

## 2. Materials and Methods

### 2.1. Housing, Animals, and Experimental Design

The study was conducted at Servei de Granges i Camps Experimentals (Universitat Autònoma de Barcelona, Bellaterra, Barcelona, Spain). All experimental procedures received prior approval from the Animal Ethics Committee (Permit No. CEEAH 3938) and were carried out in accordance with European Union Guidelines for the care and use of animals in research [16]. A total of 24 one-day-old Ross 308 chickens (bonÀrea, Verdú, Lleida, Spain) were weighed and randomly allocated to one of the two dietary treatments, with 3 chicks per cage and 4 cages per treatment. Feed and water were offered ad libitum, and animals were raised under controlled conditions of light and temperature, as recommended by the breeder.

Broiler chickens received a starter feed until day 21 and a grower-finisher diet from day 22 to day 44, both in mash form. Diets based on corn, wheat and soybean meal were formulated to comply with FEDNA requirements [17]. The two dietary treatments consisted in the basal diet non-supplemented as the control (CTR) group and the basal diet supplemented with a feed additive at 1 kg/t (DICOSAN+; DIC) supplied by Norel S.A. (Madrid, Spain). This feed additive consists of 70% sodium butyrate (56% butyric acid) partially protected by sodium salts of MCFA obtained from coconut fatty acid distillates as by-products of the oil-refining process (0.84% caprylic acid, 0.84% capric acid, 11.5% lauric acid, 3.84% myristic acid, 2.04% palmitic acid, 0.60% stearic acid, 3.72% oleic acid).

Two days before sampling, the DIC group received the feed additive containing 3% of brilliant blue FCF dye (E-133), used as an inert marker that does not have adverse effects on animal health [18] to evaluate the intestinal release of butyric acid. The amount of blue color included did not disturb the physicochemical properties of the original feed additive. Furthermore, the protection of the feed additive containing few traces of brilliant blue avoided the inhalation hazards usually associated with dyes [19].

### 2.2. Sampling and Analytical Determinations

At 44 d of age, one animal per cage was electrically stunned (electrical stunner Reference: 100523, FAF; Saint-Sernin-sur-Rance, France) and euthanized. The entire GIT was then removed and digestive contents from duodenum, jejunum, and ileum were collected. Then, jejunum (from the most distal point of duodenum to Meckel’s diverticulum) and ileum (from Meckel’s diverticulum to the ileocecal junction) were divided into 2 equal sections, named as proximal and distal. Digesta samples were kept immediately on ice to analyze the released brilliant blue. A calibration curve was performed using a total of 15 dilute reference solutions (reference solution 1.9979 mM). The absorbance was measured using a spectrophotometer (SpectraMax Plus384) at 570 nm (adapted from Suarez et al. [20]). The calibration equation obtained was Y = 1.823X + 0.109, where “Y” was the absorbance at 570 nm and “X” was the concentration, and the R^2^ was 0.995.

Digestive samples were defrosted and an aliquot of 2 g was collected to be homogenized with 3 mL of distilled water for 30 s with a vortex. The homogenized samples were centrifuged at 3000× *g* for 45 min at 4 °C while maintaining refrigeration conditions to keep intestinal enzymes in standby (adaptation of Lee et al. [21]). The supernatant solution was pipetted into Eppendorf and centrifuged for 30 min at the same conditions as before. The supernatant resulting from the second centrifugation was collected to determine its absorbance at 570 nm. The absorbance of the CTR group was used to calculate an average for each intestinal segment and establish it as blank for the absorbance of the DIC group. The concentration of brilliant blue was calculated using the calibration equation to determine the percentage of released brilliant blue. As the dye was included in the feed additive following the methodology developed to protect butyrate using sodium salts of MCFA, the concentration of brilliant blue released was considered an indicator for the intestinal butyric acid delivered.

### 2.3. Statistical Analysis

The experimental unit was the cage. Statistical analyses were performed by a linear model using software R version 3.6.0 [22]. The model included the intestinal segments as the main factor and the differences between them were tested using Tukey’s method. Differences were declared significant at *p* < 0.05. The residual standard error was used as the measure of error.

## 3. Results and Discussion

Butyric acid is a natural SCFA produced by fermentation of intestinal microbiota [3]. Thus, this SCFA can be detected in the intestine of broilers that did not receive dietary butyrate, limiting the quantification of butyric acid released from protected feed additives, in contrast to other additives. Indeed, Choi et al. [9] determined the amount of thymol used in protected form by gas chromatography flame ionization detectors after extracting this essential oil from digesta samples. As this methodology cannot be considered for a natural intestinal product, several authors have tried to quantify the delivery of butyric acid throughout the GIT using markers.

Some researchers used radioactive markers as ^14^C and ^13^C. Particularly, Smith et al. [23] added calcium ^1–14^C butyrate to unlabeled calcium butyrate used as a dietary supplement. Luminal contents were combusted, so the resultant radioactive carbon dioxide was determined to quantify the free butyric acid in the intestine. Background radioactivity was determined for each intestinal segment as blanks, and a delayed determination of butyrate was observed in broilers supplemented with protected butyrate in contrast to non-protected butyrate. However, cumulative losses of gaseous radioactivity from the metabolism crates at slaughter and during dissection limited the interpretation of the results [23]. Furthermore, some authors monitored the expiration of labeled carbon dioxide after broilers were fed and labeled ^13^C or ^14^C butyrate. Therefore, the amount of butyric acid released was determined per intestinal segment according to the GIT passage rate. However, the expiration of the labeled dioxide carbon was not as immediate as assumed, causing a delay [23,24]. Furthermore, the variable GIT passage times influenced by such things as breed, diet composition, and particle size also made the analysis of butyric acid released in the intestinal content difficult, although an indicator of stomach passage (octanoic acid) was used [24]. Thus, an alternative methodology was searched for in the present study to avoid these limitations. Consequently, a dye (brilliant blue) was used as an inert marker in the current study to evaluate the intestinal delivery of butyric acid from fat-protected feed additive.

**Figure 1:**
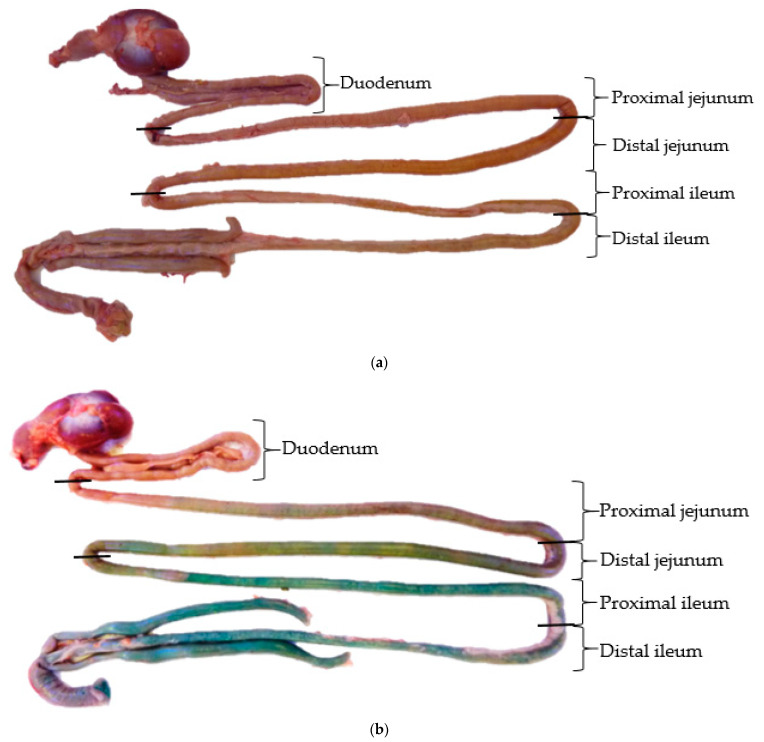
The GIT of a 44-day-old broiler: (**a**) non-supplemented (CTR); (**b**) supplemented with protected sodium butyrate containing protected brilliant blue dye (DIC).

Figure 1b shows how the GIT of a 44-day-old broiler chicken received a diet supplemented by protected sodium butyrate containing brilliant blue. The blue color was observed in the jejunum and in the posterior intestinal sections, suggesting that the gradual release started from jejunum and backward. This result is supported by Abdelli et al. [15] who observed that non-protected brilliant blue dye colored all the GIT of the broilers, whereas the blue color was only observed from the jejunum of the birds receiving brilliant blue dye protected in another feed additive. However, a quantification of brilliant blue dye liberated in the intestine was not performed in previous studies because the intestinal content makes the color turn to green by the dietary corn, and the calibration curve will no longer be correct.

In the present study, the digesta color of different non-supplemented broilers (Figure 1a) was considered as blank in order to avoid the limitations discussed in prior studies [15,21]. Therefore, the equation of the calibration curve allowed for the quantification of intestinally released brilliant blue, and consequently, butyric acid, regardless of diet composition (Table 1). It should be noted that as fats are digested and absorbed in the small intestine and butyric acid was protected by fat material in the tested feed additive, the distal segment of ileum was considered the final intestinal segment for the total release of this SCFA [25]. As is shown in Table 1, only 1.80% of butyric acid was delivered to the duodenum, and 5.74% of butyric acid was released in the proximal jejunum. The butyric acid was then delivered in the distal jejunum and proximal ileum at 15.4% and 31.1%, respectively. The remaining 45.9% of butyric acid was released in the distal ileum. Therefore, a gradual release can be defined (*p* < 0.001) as releasing minimal amounts of butyric acid in the duodenum and delivering high amounts of this SCFA to the distal ileum [15,24].

The 1.80% and 5.74% of the released butyric acid in the duodenum and proximal jejunum can be attributed to the non-protected butyric acid contained in the feed additive. In fact, according to the manufacturer, sodium butyrate was partially protected by the sodium salts of the MCFA. This proximal detection can also be explained by the anti-peristaltic movement from distal ileum [26,27]. Although some free sodium butyrate was observed in the proximal small intestine, most of the butyric acid was released in the ileum, particularly in the posterior tract. It has been hypothesized that this could be due to the major intestinal site of fatty acid absorption being the jejunum, although the ileum also plays an important role [25].

## 4. Conclusions

In summary, the qualitative (visual color) and quantitative (by spectrophotometry) results suggest that the developed modified in vivo approach is a useful strategy to evaluate the intestinal release of butyric acid presented in a protected form. In this context, the use of sodium butyrate protected by sodium salts of MCFA seems to be a promising feed additive to increase the availability of butyric acid to promote gut health due to its gradual intestinal release.

## Figures and Tables

**Table 1:** Percentage of released butyric acid in the small intestine of broiler chickens supplemented with protected sodium butyrate.

Item	Duodenum	Proximal Jejunum	Distal Jejunum	Proximal Ileum	Distal Ileum	RSE	*p*-Value
Butyric acid, %	1.80 ^d^	5.74 ^cd^	15.4 ^c^	31.1 ^b^	45.9 ^a^	4.88	<0.001

RSE = residual standard error. a–d Means within a row with different superscript significantly differ (*p* ≤ 0.05).

## Data Availability

Not applicable.
